# Incidence rates of influenza illness during pregnancy in Suzhou, China, 2015–2018

**DOI:** 10.1111/irv.12888

**Published:** 2021-07-29

**Authors:** Liling Chen, Suizan Zhou, Lin Bao, Alexander J. Millman, Zhongwei Zhang, Yan Wang, Yayun Tan, Ying Song, Pengwei Cui, Yuanyuan Pang, Cheng Liu, Jiangchun Qin, Ping Zhang, Mark G. Thompson, A. Danielle Iuliano, Ran Zhang, Carolyn M. Greene, Jun Zhang

**Affiliations:** ^1^ Suzhou Center for Disease Control and Prevention Suzhou China; ^2^ U.S. Centers for Disease Control and Prevention Atlanta GA USA; ^3^ Suzhou Municipal Hospital Suzhou China; ^4^ Wuzhong Maternal and Child Health Care Institute Suzhou China

**Keywords:** active surveillance, cohort, incidence rate, influenza, pregnant women

## Abstract

**Background:**

Data on influenza incidence during pregnancy in China are limited.

**Methods:**

From October 2015 to September 2018, we conducted active surveillance for acute respiratory illness (ARI) among women during pregnancy. Nurses conducted twice weekly phone and text message follow‐up upon enrollment until delivery to identify new episodes of ARI. Nasal and throat swabs were collected ≤10 days from illness onset to detect influenza.

**Results:**

In total, we enrolled 18 724 pregnant women median aged 28 years old, 37% in first trimester, 48% in second trimester, and 15% in third trimester, with seven self‐reported influenza vaccination during pregnancy. In the 18‐week epidemic period during October 2015 to September 2016, influenza incidence was 0.7/100 person‐months (95% CI: 0.5–0.9). In the cumulative 29‐week‐long epidemic during October 2016 to September 2017, influenza incidence was 1.0/100 person‐months (95% CI: 0.8–1.2). In the 11‐week epidemic period during October 2017 to September 2018, influenza incidence was 2.1/100 person‐months (95% CI: 1.9–2.4). Influenza incidence was similar by trimester. More than half of the total influenza illnesses had no elevated temperature and cough. Most influenza‐associated ARIs were mild, and <5.1% required hospitalization.

**Conclusions:**

Influenza illness in all trimesters of pregnancy was common. These data may help inform decisions regarding the use of influenza vaccine to prevent influenza during pregnancy.

## 1 INTRODUCTION

Pregnant women are known to be at risk for severe influenza disease and influenza‐associated morbidity and mortality.^1^, ^2^, ^3^, ^4^ During the 2009 influenza pandemic, influenza illnesses were observed to be associated with complications during pregnancy including maternal mortality and loss of pregnancy.^5^, ^6^, ^7^, ^8^ The World Health Organization (WHO) and national public health authorities have recommended vaccination, the best tool for preventing influenza illness and complications associated with infection, for high risk groups including for pregnant women.^9^, ^10^ However, despite recommendation from the Chinese Center for Disease Control and Prevention (China CDC),^11^ seasonal influenza vaccination coverage during pregnancy in China is less than 1%.^12^

In China, there are significant barriers to promoting seasonal influenza vaccination in pregnant women including an absolute contraindication for this population in the Chinese Pharmacopeia until the latest revision in 2020, healthcare worker reluctance to recommend this vaccination to pregnant women, and women's concerns about vaccine safety or lack of awareness of vaccination.^13^, ^14^, ^15^, ^16^ Knowledge of influenza infection during pregnancy is also limited. Previous studies found that only 20% of surveyed pregnant women were aware that influenza infection could cause serious harm during pregnancy and 61% reported an interest in learning about prevention and control of influenza.^17^

Data are needed to evaluate burden of influenza virus infection during pregnancy in China, to enhance risk communication and to inform future prevention and control measures including increasing influenza vaccine uptake within this population. We conducted active surveillance among pregnant women in Suzhou, China, to assess influenza incidence in a population at risk for influenza disease.

## 2 METHODS

### 2.1 Study design and participants

From October 2015 to September 2018, we conducted active surveillance for laboratory‐confirmed influenza‐associated acute respiratory illness (ARI) among pregnant women in Suzhou. Suzhou is a city located in eastern China with an estimated population of approximately 10 million in 2015.^18^ Influenza surveillance data from Suzhou and similar geographic areas have demonstrated semi‐annual seasonal patterns with peak activity typically occurring in January–February and June–August.^19^

The enrollment methodology and description of the cohort profile of the China Respiratory Illness Surveillance among Pregnant women (CRISP) have been published previously.^12^ In brief, from October 2015 to September 2018, nurses consented and enrolled pregnant women in different trimesters of pregnancy (first trimester defined as 1–12 weeks gestation, second as 13–26 weeks gestation, and third as 27 weeks to gestation) from two prenatal care facilities that provide prenatal care services to 17% of women residing in Suzhou during pregnancy^20^ and one pre‐marriage health center. Prenatal care facilities were selected as enrollment sites because more than 95% of pregnant women who had a live birth or stillbirth delivery after 28 weeks gestation in Suzhou had made at least one prenatal care visit at a prenatal care facility and because pregnancy status would be confirmed by ultrasound as part of routine care.^20^ We selected a pre‐marriage health center, which offers pregnancy testing to couples, as an enrollment site to identify women who may have been early in their pregnancies. Pregnant women who lived in and planned to deliver in Suzhou were considered eligible for enrollment. Pregnant women who sought non‐routine prenatal care such as confirmation of low progesterone and threatened miscarriage were excluded.

### 2.2 Enrollment of the annual cohort

**FIGURE 1:**
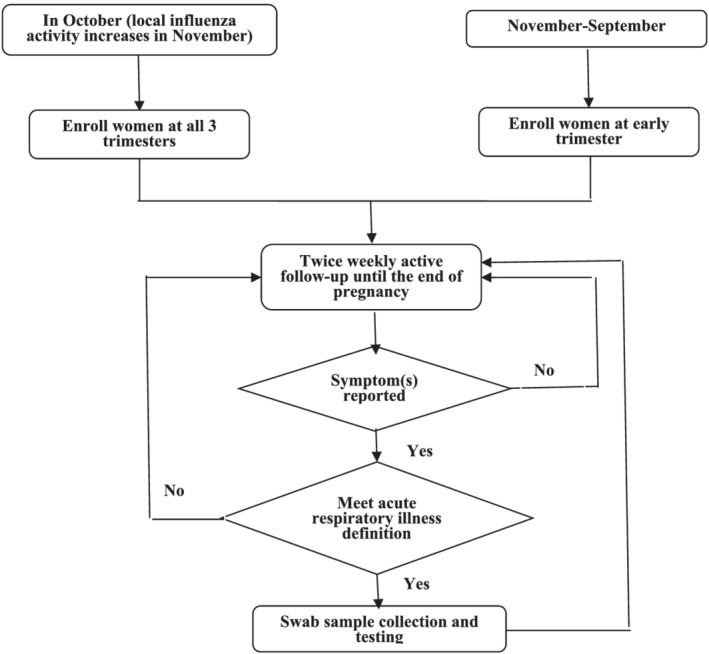
Flow chart of enrollment and follow‐up of the annual cohorts of China Respiratory Illness Surveillance among Pregnant Women (CRISP), Suzhou, 2015/2016–2017/2018 seasons. After enrolling pregnant women, study nurses conducted twice weekly follow‐up starting at the enrollment date until the delivery date or the end of pregnancy/loss of pregnancy with one phone call and one WeChat text message (a free, instant messaging application widely used in China) to identify episodes of acute respiratory illness (ARI). An ARI episode was defined as ≥1 respiratory symptom (cough, sore throat, stuffy nose, chest pain, and difficulty breathing) and ≥1 systemic symptom (feverish, temperature ≥38°C, chills, and headache) or ≥2 respiratory symptoms

Study nurses enrolled pregnant women into annual cohorts from October to September of each study year that was defined as the influenza season in the national influenza surveillance protocol.^21^ Most individuals in each cohort were enrolled before November. However, to ensure enrollment of adequate numbers of first trimester pregnant women and to ensure sufficient numbers of pregnant women to follow during the summer, we continuously enrolled first trimester pregnant women from the October–September period, which allowed us continuous observations throughout the year (Figure 1).

Upon enrollment, study nurses conducted face‐to‐face interviews using a structured questionnaire to collect data on the participant's demographics, health conditions, pregnancy‐associated conditions, health behaviors, social behaviors, and self‐reported influenza vaccination status. For health conditions, chronic diseases referred to any medical problem diagnosed by a doctor or other health care provider before pregnancy that lasted for at least 6 months such as diabetes, asthma, heart disease, or cancer. Pregnancy‐associated conditions referred to a newly developed condition identified during pregnancy by enrollment such as gestational diabetes or high blood pressure. Health and social behaviors included dietary supplement intake, smoking, alcohol, and self‐reported changes in working hours and social activities.

### 2.3 Follow‐up for ARI episodes and laboratory testing

After enrolling pregnant women in active surveillance, study nurses conducted twice weekly follow‐up starting at the enrollment date until the delivery date or the end of pregnancy/loss of pregnancy with phone call and WeChat text message (a free, instant messaging application widely used in China) alternatively to identify episodes of ARI. An ARI episode was defined as ≥1 respiratory symptom (cough, sore throat, stuffy nose, chest pain, and difficulty breathing) and ≥1 systemic symptom (feverish, temperature ≥38°C, chills, and headache) or ≥2 respiratory symptoms. When a current illness or recent illness was identified, the study nurse used a standardized questionnaire to collect data on illness onset date, symptoms, and hospital admission. Participants reporting ARI were encouraged to visit the study hospital's respiratory illness department (ambulatory clinics) or offered a house call for testing to ensure specimen collection ideally within 24 h but up to a maximum 10 days from illness onset. Study nurses collected both a nasal and a throat swab with double‐headed disposable virus sampling tube (YOCON Biology Technology Company, Beijing). Respiratory specimens were transported to the Suzhou CDC laboratory within 24 h of collection at 4°C and were analyzed using real‐time reverse transcription polymerase chain reaction to test for influenza virus subtype/lineage.

### 2.4 Data analysis

The cohorts were considered open, and eligible participants could be enrolled at any time during the study period. An enrolled participant was considered to have complete data if she completed the enrollment interview, responded to weekly surveillance contacts by study nurses until delivery or termination of pregnancy, and had perinatal records available for review. Those who were lost to follow‐up (no response to any contact for at least two consecutive weeks despite multiple attempts), voluntarily withdrew, or left Suzhou before the end of pregnancy only contributed the person‐time for which there was available data. If a pregnant woman was enrolled in an annual cohort but delivered after the end of the surveillance year, the person‐time was split between the two surveillance years. For example, if a woman was enrolled at week 10 of pregnancy and 15 weeks of her remaining pregnancy occurred in one surveillance year and the other 15 occurred in the following surveillance year, we considered 15 weeks of person‐time to have occurred in surveillance year 1 and 15 weeks of person‐time to have occurred in surveillance year 2. To streamline cohort management and reporting, the full analytic population for each surveillance year included those contributing person‐time from the previous surveillance year if applicable.

We calculated incidence rate over the influenza epidemic periods of each influenza surveillance year. The start of each influenza epidemic period was defined as the first day of three consecutive influenza reporting weeks in which the percentage of specimens testing positive for any influenza virus infection was higher than 5%. The end of each influenza epidemic period was defined as the day before the first of three consecutive influenza reporting weeks in which the percentage of specimens testing positive for influenza was below 5%.^22^ In the influenza incidence rates calculation, illness episodes were considered as distinct episodes if they occurred at least 2 weeks apart, from the end of symptoms for one episode to the onset of any new symptom, and the population at risk was defined as women pregnant for 2 weeks or more during the epidemic period.

Our primary outcome was the influenza incidence identified through ARI screening. For secondary outcomes, we evaluated the influenza incidence using influenza‐like illness (ILI) case definitions for screening. ILI was defined as a measured temperature ≥38°C and cough/sore throat.

We described demographic, clinical, and knowledge and practices related to influenza vaccination. Chi‐square or Fisher exact tests were used to compare proportions where appropriate. Incidence rate was calculated as the number of new cases per 100 person‐month. Since the total number of missed swabs was minimal, those with missing swabs contributed person time but did not contribute to the total number of influenza positive cases; 95% confidence interval (CI) for person‐month rate was simulated with bootstrapping. Fisher exact test was used to compare person‐month rates. All tests were two sided, and a *p* < 0.05 was considered as statistically significant. R version 3.5.3 was used for statistical analysis.

## 3 RESULTS

### 3.1 Cohort characteristics

**FIGURE 2:**
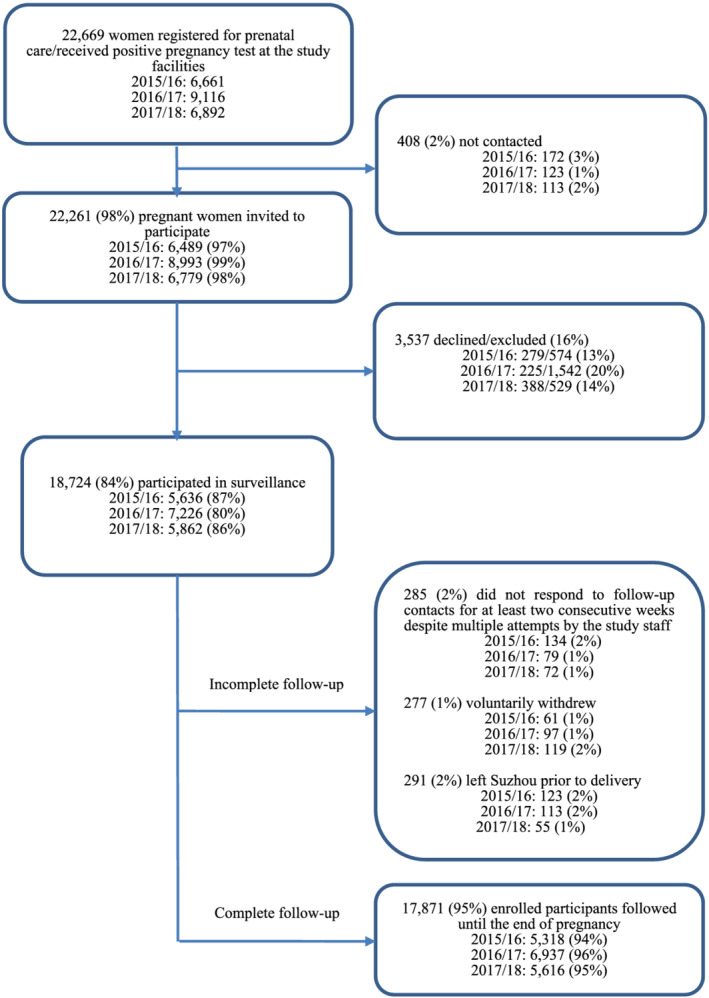
Profile of the annual cohorts of China Respiratory Illness Surveillance among Pregnant Women (CRISP), Suzhou, 2015/2016–2017/2018 seasons. Among 2016/2017 cohort, 1873 women continued follow up from 2015/2016 season; among 2017/2018 cohort, 2342 women continued follow up from 2016/2017 season

Among the women registered for prenatal care or with positive pregnancy tests in study facilities, we had high contact rates (reached) of 97% (6489/6661), 99% (8993/9116), and 98% (6779/6892) over three cohorts, respectively. Among those reached, 84% (18 724/22 261) participated in the prospective cohorts with participation rates of 87% (5636/6489) in year 1 cohort, 80% (7226/8993) in year 2 cohort, and 86% (5862/6779) in year 3 cohort. Among participants, 95% (17 871/18 724) had complete follow‐up information until delivery with minimal variability by cohort year. Most participants who did not complete follow‐up either left Suzhou (2%, 291/18 724), voluntarily withdrew (1%, 277/18 724), or were lost to follow‐up (2%, 285/18 724) (Figure 2).

Among all 18 724 enrolled participants, the median age was 28 years old, and 73% reported college or higher level of education. At enrollment, 37% were in the first trimester, 48% were in the second trimester, and 15% were in the third trimester. Among participants, 3% reported a previously diagnosed chronic disease, and 1% reported a pregnancy‐associated medical condition by enrollment. Among the 18 724 pregnant women, 2918 (18%) who reported not working in the week prior to enrollment, pregnancy was the self‐reported primary reason among 86% for not working. Of all participants, 40% reported decreased working hours during pregnancy, 52% reported a reduction in general social interactions, and 57% reported reduced social activities with friends during pregnancy (Table 1).

**TABLE 1:** Characteristics of the annual cohorts of China Respiratory Illness Surveillance among Pregnant Women (CRISP), Suzhou, 2015/2016–2017/2018 seasons

	2015/2016 cohort (*N* = 5636)	2016/2017 cohort (*N* = 7226)	2017/2018 cohort (*N* = 5862)	Overall (*N* = 18 724)
Demographics				
Age				
<25 y	937/5636 (17)	1014/7216 (14)	818/5862 (14)	2769/18 714 (15)
25–29 y	3183/5636 (56)	3747/7216 (52)	3033/5862 (52)	9963/18 714 (53)
30–34 y	1181/5636 (21)	1798/7216 (25)	1488/5862 (25)	4467/18 714 (24)
≥35 y	335/5636 (6)	657/7216 (9)	523/5862 (9)	1515/18 714 (8)
Han ethnicity	5494/5549 (97)	7119/7188 (99)	5764/5824 (98)	18 377/18 561 (99)
Suzhou permanent residency registration	2910/5587 (52)	3890/7193 (54)	2997/5828 (51)	9797/18 608 (53)
College or higher education	4094/5619 (73)	5340/7196 (74)	4229/5828 (72)	13 663/18 643 (73)
Trimester at enrollment				
First (<13 gestational weeks)	1407/5636 (25)	3114/7226 (43)	2408/5862 (41)	6929/18 724 (37)
Second (13–27 gestational weeks)	3052/5636 (54)	3271/7226 (45)	2659/5862 (45)	8982/18 724 (48)
Third (≥28 gestational weeks)	1177/5636 (21)	841/7226 (12)	795/5862 (14)	2813/18 724 (15)
Medical history				
Underlying chronic disease^a^	153/5636 (3)	170/7226 (2)	149/5862 (3)	472/18 724 (3)
Newly diagnosed condition during pregnancy^b^	98/5636 (2)	76/7226 (1)	91/5862 (2)	265/18 724 (1)
Health behaviors				
Take vitamins, minerals or other dietary supplements before pregnancy	2437/5514 (44)	3905/7126 (55)	2865/5759 (50)	9207/18 399 (50)
Take vitamins, minerals or other dietary supplements during pregnancy	3956/5521 (72)	5523/7148 (77)	4412/5780 (76)	13 891/18 449 (75)
Have ever used or taken any medication during pregnancy	537/5490 (10)	495/7151 (7)	464/5794 (8)	1496/18 435 (8)
Ever smoked	52/5606 (0.9)	38/7175 (0.5)	27/5825 (0.5)	117/18 606 (0.6)
During the past 7 days, a household member smoked tobacco in your presence	366/5636 (6)	221/7226 (3)	220/5862 (4)	807/18 724 (4)
During the past 7 days, a colleague smoked tobacco in your presence	213/5636 (4)	142/7226 (2)	114/5862 (2)	469/18 724 (3)
During the past month, consumed an alcoholic beverage at least once (including beer, wine, and other liquor)	59/5590 (1)	65/7175 (1)	43/5825 (1)	167/18 590 (1)
Social behaviors				
Not working in the entire prior week	1109/4931 (22)	952/6211 (15)	857/5065 (17)	2918/16 207 (18)
Pregnancy as the reason for being not working	941/1065 (88)	813/945 (86)	715/858 (83)	2469/2868 (86)^c^
Reduced work hours since aware of pregnancy	1988/4738 (42)	2534/6204 (41)	1930/5065 (38)	6452/16 007 (40)
Reduced general social interactions since pregnancy	2804/5588 (50)	3846/7173 (54)	3040/5824 (52)	9690/18 585 (52)
Reduced social activities with friend	3528/5595 (63)	4107/7177 (57)	3042/5825 (52)	10 677/18 597 (57)
Influenza vaccination				
Ever heard of influenza vaccine	3897/5636 (69)	4562/7226 (63)	3054/5862 (52)	11 513/18 724 (61)
Vaccinated in the prior 1 year	44/5636 (0.8)	32/7226 (0.4)	32/5862 (0.5)	108/18 724 (0.6)
Vaccinated during pregnancy	2/5636 (0.0)	1/7226 (0.0)	4/5862 (0.0)	7/18 724 (0.0)
Any vaccination in family members	22/5636 (0.4)	21/7226 (0.3)	74/5862 (1.3)	117/18 724 (0.6)

*Note*: Data were *n*/*N* (%).

^a^ Chronic disease refers to any medical problem diagnosed by a doctor or other health care provider before pregnancy that lasted for at least six months such as diabetes, asthma, heart disease, or cancer.

^b^ Newly diagnosed condition during pregnancy: pregnancy induced new health problems or diseases such as diabetes, high blood pressure diagnosed during pregnancy.

^c^ Changes in denominator reflect missing data or refusal to answer.

Of the 18 724 women, 61% reported knowing of influenza vaccination. Only 7/18 724 (0%) reported that they had been vaccinated during pregnancy. Only 0.6% reported influenza vaccination in the prior year and 0.6% reported influenza vaccination among family members in the prior year. The proportion vaccinated during pregnancy did not differ significantly across cohorts (Table 1).

### 3.2 Influenza incidence

**FIGURE 3:**
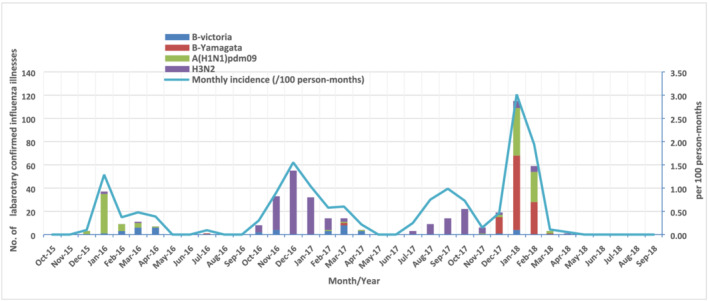
Number of laboratory‐confirmed influenza illnesses by subtype/lineage and monthly incidence in the annual cohorts of China Respiratory Illness Surveillance among Pregnant Women (CRISP), Suzhou, 2015/2016–2017/2018 seasons

From October 2015 to September 2016, we followed participants for a total of 22 125 person‐months, with an average of 3.9 months for each participant and a standard deviation (SD) of 2.5 months. In 2015/2016, we collected samples for 83% of the 1429 ARIs reported. Of those, 67 (6%) were influenza positive. The influenza epidemic period for this season was 18 weeks from Week 53, 2015, to Week 17, 2016, with an influenza incidence of 0.7/100 person‐months (95% CI: 0.5–0.9), and A(H1N1)pdm09 virus most frequently detected (Table 2, Figure 3).

**TABLE 2:** Influenza incidence during the epidemic periods^a^ in the annual cohorts of China respiratory illness surveillance among pregnant women (CRISP), Suzhou, 2015/2016–2017/2018 seasons (per 100 person‐months)

	2015/2016		2016/2017		2017/2018		Overall incidence	
	Rate (95% CI)	*p*	Rate (95% CI)	*p*	Rate (95% CI)	*p*	Rate (95% CI)	*p*
ARI influenza by age at enrollment								
<25 y	0.6 (0.3, 1.0)	REF	0.8 (0.4, 1.3)	REF	2.4 (1.6, 3.2)	REF	1.1 (0.8, 1.5)	REF
25–29y	0.6 (0.4, 0.8)	1.000	1.1 (0.9, 1.3)	0.362	2.0 (1.6, 2.4)	0.559	1.2 (1.0, 1.3)	0.885
30–34y	1.0 (0.5, 1.4)	0.354	0.9 (0.6, 1.2)	0.960	2.1 (1.6, 2.7)	0.741	1.3 (1.0, 1.5)	0.618
35y	0.4 (0.0, 1.0)	0.864	1.1 (0.6, 1.7)	0.539	2.3 1.2, 3.4	1.000	1.3 (0.9, 1.7)	0.613
ARI influenza by education level								
Junior high school (9th grade) or lower	0.8 (0.3, 1.5)	REF	0.9 (0.3, 1.6)	REF	2.7 (1.7, 3.8)	REF	1.5 (1.0, 2.0)	REF
Senior high school (12th)	0.7 (0.3, 1.1)	0.897	1.3 (0.9, 1.7)	0.504	2.6 (1.9, 3.4)	0.948	1.5 (1.2, 1.8)	1.000
College or higher	0.7 (0.5, 0.9)	0.732	1.0 (0.8, 1.1)	1.000	1.9 (1.6, 2.3)	0.181	1.1 (1.0, 1.2)	0.100
Syndrome								
ARI^b^	0.7 (0.5, 0.9)	REF	1.0 (0.8, 1.2)	REF	2.1 (1.9, 2.4)	REF	1.2 (1.1, 1.3)	REF
ILI^c^	0.3 (0.2, 0.4)	**<0.001**	0.3 (0.2, 0.4)	**<0.001**	0.6 (0.4, 0.7)	**<0.001**	0.4 (0.3, 0.5)	**<0.001**

Abbreviations: CI, confidence interval; NA, not applicable; REF, reference.

^a^ The start of each influenza epidemic period was defined as the first day of three consecutive influenza reporting weeks in which the percentage of specimens testing positive for any influenza virus infection was higher than 5%. The end of each influenza epidemic period was defined as the day before the first of three consecutive influenza reporting weeks in which the percentage of specimens testing positive for influenza was below 5%.

^b^ ARI: acute respiratory illness, defined as ≥1 respiratory symptom (cough, sore throat, stuffy nose, chest pain, and difficulty breathing) and ≥1 systemic symptom (feverish, temperature ≥38°C, chills, headache); or ≥2 respiratory symptoms with onset within the last 10 days.

^c^ ILI: influenza‐like illness, defined as a measured temperature ≥38°C and cough/sore throat with onset within the last 10 days.

From October 2016 to September 2017, we observed a total of 25 775 person‐months, with an average of 3.6 months for each participant (SD 2.6). In 2016/2017, 98% of 2637 ARIs reported had samples collected. Of those, 185 samples (7%) were influenza positive. The influenza incidence during the total 29‐week long epidemic with two separate periods from Week 47, 2016, to Week 13, 2017, and from Week 30, 2017, to Week 39, 2017, was 1.0/100 person‐months (95% CI: 0.8–1.2), and A(H3N2) virus was most frequently detected (Table 2, Figure 3).

From October 2017 to September 2018, we observed a total of 27 102 person‐months, with an average of 4.6 months for each participant (SD 2.2). In 2017/2018, of 2402 ARI reported, 96% had samples collected. Of those, 225 samples (10%) were influenza positive. The influenza incidence during the 11‐week epidemic period, from Week 52, 2017, to Week 10, 2018, was 2.1/100 person‐months (95% CI:1.9–2.4), with B virus Yamagata lineage most frequently detected (Table 2, Figure 3).

### 3.3 Factors associated with the incidence estimates

No significant difference in influenza incidence was observed by age or education level. Influenza incidence varied significantly when using different surveillance case definitions. Using ILI as the surveillance case definition had significantly lower influenza incidence (0.4 per 100 person‐months) than using ARI (1.2 per 100 person‐months) in overall of the epidemic periods (*p* < 0.001). The findings regarding demographic characteristics' and case definitions' difference in influenza illnesses were consistent over the entire study period (Table 2). These findings were also consistent when expanding the observation outside of the epidemic period (data not shown).

In the overall cohort, ARI incidence decreased significantly in the second trimester (10.0 per 100 person months) and in the third trimester (7.6 per 100 person‐months) in comparison with the first trimester (21.0 per 100 person‐months) during the epidemic periods (Table 3). This observation was consistent in all three cohorts. However, in the overall cohort, we found no significant differences in the influenza incidence by trimester, which was 1.4 per 100 person‐months during the first trimester and 1.2 per 100 person‐months during both the second and third trimesters. Within the three cohorts, influenza incidence did not differ by trimester in the 2015/2016 and 2016/2017 cohorts, while in the 2017/2018 cohort, the influenza incidence for women in the third trimester (2.0 per 100 person‐months) was significantly lower compared with the influenza incidence in the first trimester (3.8 per 100 person‐months). As is shown in Table 3, among influenza‐associated ARIs in the three annual cohorts, three of 66 (4.5%), eight of 158 (5.1%), and three of 187 (1.6%) required hospitalization during epidemic period. Overall, compared with first (0%) trimester and second (0.7%) trimester, influenza‐associated ARIs in the third (5.4%) trimester were more frequently hospitalized.

**TABLE 3:** Acute respiratory illness (ARI),^a^ influenza illness and influenza hospitalization during the epidemic periods^b^ by the trimester of symptom onset in the annual cohorts of China respiratory illness surveillance among pregnant women (CRISP), Suzhou, 2015/2016–2017/2018 seasons

	ARI incidence (per 100 person‐months, 95% CI)	p	Influenza incidence (per 100 person‐months, 95% CI)	p	Influenza hospitalization, *n*/*N* (% of influenza ARI)	*p*
Overall					**13/411 (3.2)**	
First	21.0 (19.5, 22.6)	REF	1.4 (1.0, 1.9)	REF	0/39 (0.0)	REF
Second	10.0 (9.5, 10.5)	**<0.001**	1.2 (1.0, 1.4)	0.419	1/151 (0.7)	
Third	7.6 (7.2, 7.9)	**<0.001**	1.2 (1.0, 1.3)	0.333	12/221 (5.4)	**0.005**
2015/2016					3/66 (4.5)	
First	17.6 (15.3, 20.0)	REF	0.7 (0.2, 1.3)	REF	0/6 (0.0)	REF
Second	8.3 (7.4, 9.4)	**<0.001**	0.7 (0.4, 1.0)	1.000	0/22 (0.0)	
Third	6.3 (5.7, 6.9)	**<0.001**	0.7 (0.5, 0.9)	0.941	3/38 (7.9)	0.256
2016/2017					8/158 (5.1)	
First	22.8 (20.6, 24.9)	REF	1.2 (0.6, 1.8)	REF	0/17 (0.0)	REF
Second	11.2 (10.3, 11.8)	**<0.001**	1.0 (0.7, 1.2)	0.527	0/59 (0.0)	
Third	8.4 (7.8, 9.0)	**<0.001**	1.0 (0.8, 1.3)	0.728	8/82 (9.8)	**0.007**
2017/2018					3/187 (1.6)	
First	22.6 (18.6, 26.7)	REF	3.8 (2.1, 5.6)	REF	0/16 (0.0)	REF
Second	9.5 (8.5, 10.5)	**<0.001**	2.1 (1.7, 2.6)	0.063	1/70 (1.4)	
Third	7.7 (7.0, 8.4)	**<0.001**	2.0 (1.6, 2.4)	**0.032**	2/101 (2.0)	1.000

Abbreviations: CI, confidence interval; REF, reference.

^a^ Acute respiratory illness (ARI) defined as ≥1 respiratory symptom (cough, sore throat, stuffy nose, chest pain, and difficulty breathing) and ≥1 systemic symptom (feverish, temperature ≥38°C, chills, headache) or ≥2 respiratory symptoms with onset within the last 10 days.

^b^ The start of each influenza epidemic period was defined as the first day of three consecutive influenza reporting weeks in which the percentage of specimens testing positive for any influenza virus infection was higher than 5%. The end of each influenza epidemic period was defined as the day before the first of three consecutive influenza reporting weeks in which the percentage of specimens testing positive for influenza was below 5%.

## 4 DISCUSSION

From 2015 to 2018, influenza incidence during epidemic periods in pregnant women varied by year ranging from 0.7 to 2.1 per 100 person‐months. Influenza vaccination during pregnancy was rare although approximately half of the women enrolled reported social distancing behavioral changes during pregnancy. Influenza incidence was similar across trimesters. Using ILI as a case definition missed more than half of the total influenza illnesses compared with broader ARI criteria. Over the three cohorts, 1.6–5.1% of influenza‐associated ARI cases required hospitalization.

Prior to this study, there were limited data on influenza incidence and epidemiology of infection during pregnancy in China.^23^ By following cohorts of pregnant women over multiple seasons using a sensitive case definition and highly sensitive and specific molecular diagnostics, we found that during influenza epidemic periods in 2015–2018 in Suzhou, women were frequently infected with influenza during pregnancy and in some cases had illness requiring hospitalization. Our estimated influenza incidence rates were similar with a prospective cohort study in three middle income countries that showed women had a 0.7–0.9% risk of influenza per month of pregnancy during the 2017–2018 influenza seasons.^24^ Our incidence estimates were higher than influenza incidence rates observed in influenza vaccination clinical trials in other low and middle income countries.^25^, ^26^, ^27^, ^28^ One reason for this difference was that unlike some other studies, our case definition included non‐febrile influenza illnesses. Requiring the presence of measured fever in the case definition as with ILI substantially reduced the number of influenza‐associated illness detected during pregnancy in Suzhou. This finding of lack of febrile illness among influenza infected pregnant patients is consistent with previously described findings from the United States in this population.^29^ Thus, our study, the first to describe influenza illness during pregnancy in China, also demonstrated the importance of including non‐febrile illness to identify influenza infection and accurately estimate symptomatic influenza incidence in this population.

Although we found that the incidence of ARI during pregnancy varied by trimester, in our cohort, influenza incidence was similar across trimesters. Studies from the United States during the 2009 influenza pandemic described a higher frequency of hospitalization among pregnant women with influenza virus illness in the third trimester.^8^, ^30^ Studies evaluating seasonal influenza hospital admission and rates of influenza outpatient visits in pregnant women have not observed differences by trimester.^31^, ^32^ Our study's active surveillance of community‐dwelling pregnant women allowed us to develop a complete picture of ARI and influenza illness during pregnancy by trimester and, unlike prior studies, did not rely on participant health‐seeking or clinician behavior for case identification.^23^, ^30^, ^31^, ^32^ In our study, risk of influenza virus infection among pregnant women was similar throughout all trimesters of pregnancy, underscoring the importance of implementing measures such as influenza vaccination to prevent influenza‐associated illness during all trimesters.

Applying the overall cohort influenza incidence rates during pregnancy of our study to a 12‐week‐long epidemic period, our result is comparable with the estimates in unvaccinated adults aged 18–64 years based on a modeling study from the United States, which estimated a cumulative seasonal incidence of symptomatic influenza of 4.3%–7.9% in seasons of moderate severity.^33^ Our findings show that women during pregnancy had similar risk of influenza illness compared with the general adult population. Furthermore, we found that only 1.6–5.1% of influenza‐associated ARIs during pregnancy in our cohort led to hospitalization. However, women during pregnancy had a disproportionately higher hospitalization burden compared with the general population in a U.S. study, which found a 0.7% case‐hospitalization ratio (183 320 hospitalization in total 27 168 060 cases) in adults aged 20–64 years.^34^ Interestingly, although the 2017/2018 cohort had the highest proportion of influenza‐associated ARI of the three surveillance years, the influenza‐associated ARI hospitalization proportion was the lowest. As the 2017/2018 influenza season in China had the highest proportion of ILI since the 2009 pandemic,^35^ it is possible that pregnant women without underlying conditions were more like to be treated as outpatients in the setting of increased demand for hospital beds.^36^

Few participants in our cohort reported receiving an influenza vaccination during their pregnancy or in the year prior to their pregnancy. This finding is consistent with other published data from China, which showed that 0.2% of participants reported influenza vaccination within the last 12 months, and none during pregnancy.^12^, ^13^ During the time of these studies, pregnancy was listed as a contraindication for seasonal influenza vaccination in the Chinese Pharmacopeia and the vaccine package insert.^15^ Although removed from the China pharmacopeia in 2020, this contraindication continues to be listed on the package insert. Despite the lack of influenza vaccination, 40–60% of participants reported social distancing behavioral modifications including reduction in work hours and social interactions during pregnancy. While there may be reasons beyond influenza prevention that our population engaged in these behavioral changes, a previous Knowledge, Attitudes, and Practices study among pregnant women in China found that barriers to influenza vaccination included low perceived susceptibility to influenza and preference for non‐pharmaceutical interventions to prevent infections.^37^ Our study demonstrates that despite behavioral modifications and perceptions, women remain susceptible to influenza virus infection during pregnancy and illness episodes may require hospitalization in even otherwise healthy individuals. Our findings highlight the importance of educating pregnant women on the benefits of influenza vaccination and addressing barriers to vaccinating pregnant women in China.

Our study had several strengths. First, this prospective cohort used laboratory‐confirmed influenza as the outcome allowing us to identify respiratory disease incidence specific to influenza. Second, the study cohort had high participation and sample collection rates compared with similar studies,^38^ which reduced the likelihood of selection bias. Twice weekly follow‐up contacts facilitated the rapid detection of illness episodes and aided in sampling respiratory viruses close to illness onset. Third, use of prenatal care facilities to recruit pregnant women enabled easy enrollment of the target population. Finally, the use of WeChat, a popular social mobile application, for active surveillance likely contributed to the high contact and retention rates.^12^

This study was subject to several limitations. First, our surveillance case definitions do not capture atypical and asymptomatic manifestations of influenza infection. Second, our cohort only describes influenza incidence during pregnancy, so we are unable to compare the findings from this population to other high‐risk populations or healthy women of child‐bearing age. Finally, Suzhou is an economically developed city in China, and the participants in our cohort were older and had higher education levels compared to pregnant women in Suzhou overall^12^; our study population may not be representative of pregnant women in rural areas of Jiangsu Province or other provinces in China.

## 5 CONCLUSIONS

Influenza illnesses in all trimesters of pregnancy were common in Suzhou, China, and we found that non‐febrile influenza illnesses were more frequent than febrile influenza illnesses during pregnancy. Pregnant woman in China are not getting vaccinated for seasonal influenza, and other studies have shown that barriers to vaccination include pregnancy listed as a contraindication in package insert, self‐perceptions that they are not at risk of infection and healthcare worker reluctance to vaccinating this population.^16^ These data can support risk communication and may help promote effective prevention and control measures for influenza including vaccination during pregnancy.

## CONFLICT OF INTERESTS

No conflict of interest declared.

## ETHICS STATEMENT

This study was approved by the Institutional Review Board (IRB) of Jiangsu Provincial Center for Disease Control and Prevention and the United States (U.S.) Centers for Disease Control and Prevention relied upon the Jiangsu Provincial IRB.

## AUTHOR CONTRIBUTIONS

**Liling Chen:** Data curation; formal analysis; methodology; project administration; software; supervision; visualization. **Suizan Zhou:** Conceptualization; methodology; project administration; validation; visualization. **Lin Bao:** Data curation; investigation; project administration; supervision. **Alexander J. Millman:** Formal analysis; methodology; validation; visualization. **Zhongwei Zhang:** Data curation; investigation; resources; supervision; visualization. **Yan Wang:** Data curation; investigation; resources; supervision; visualization. **Yayun Tan:** Data curation; formal analysis. **Ying Song:** Methodology; validation; visualization. **Pengwei Cui:** Data curation; formal analysis. **Yuanyuan Pang:** Data curation; investigation; supervision. **Cheng Liu:** Data curation; investigation; supervision. **Jiangchun Qin:** Data curation; investigation; supervision. **Ping Zhang:** Data curation; investigation; supervision. **Mark G. Thompson:** Formal analysis; methodology; visualization. **A. Danielle Iuliano:** Formal analysis; methodology; visualization. **Ran Zhang:** Methodology; project administration; validation; visualization. **Carolyn M. Greene:** Conceptualization; methodology; validation; visualization. **Jun Zhang:** Funding acquisition; methodology; project administration; software; visualization.

## DISCLAIMER

The findings and conclusions in this report are those of the authors and do not necessarily represent the official position of the Centers for Disease Control and Prevention.

### PEER REVIEW

The peer review history for this article is available at https://publons.com/publon/10.1111/irv.12888.

## Data Availability

The data that support the findings of this study are available from the corresponding author upon reasonable request.
